# Combination of ultrasound and rtPA enhances fibrinolysis in an *In Vitro* clot system

**DOI:** 10.1371/journal.pone.0188131

**Published:** 2017-11-16

**Authors:** Julia Masomi-Bornwasser, Philipp Winter, Hendrik Müller-Werkmeister, Susanne Strand, Jochem König, Oliver Kempski, Florian Ringel, Sven R. Kantelhardt, Naureen Keric

**Affiliations:** 1 Department of Neurosurgery, University Medical Center of the Johannes Gutenberg University, Mainz, Germany; 2 First Department of Internal Medicine I, University Medical Center of the Johannes Gutenberg University, Mainz, Germany; 3 Institute of Medical Biostatistics, Epidemiology and Informatics (IMBEI), University Medical Center of the Johannes Gutenberg University, Mainz, Germany; 4 Institute of Neurosurgical Pathophysiology, University Medical Center of the Johannes Gutenberg University, Mainz, Germany; Monash University, AUSTRALIA

## Abstract

**Background:**

Catheter-based lysis with recombinant tissue plasminogen activator (rtPA) is a well-established therapy for spontaneous intracerebral hemorrhage (ICH). The effectiveness of this therapy can be increased with ultrasound, but the optimal conditions are not yet clearly established. Using a novel in vitro system of blood clots previously developed by our group, we investigated various parameters of intralesional sonothrombolysis using an endosonography catheter in combination with rtPA.

**Methods:**

Standardized human blood clots were equipped with a drainage catheter and weighed before and after 4 treatments: control (drainage only), rtPA only, ultrasound only and the combination of rtPA+ultrasound. The effectiveness of ultrasound was further analysed in terms of optimal frequency, duration and distance to the probe. Temperature and acoustic peak rarefaction pressure (APRP) were assessed to analyse potential adverse effects and quantify lysis. Histo-morphological analysis of the treated clots was performed by H&E staining and confocal laser scanning microscopy using fluorescent fibrinogen.

**Results:**

The combined treatment rtPA+ultrasound achieved the highest lysis rates with a relative weight of 30.3%±5.5% (p**≤**0.0001) compared to all other groups. Similar results were observed when treating aged clots. Confocal fluorescent microscopy of the treated clots revealed a rarefied fibrin mesh without cavitations. No relevant temperature increase occurred (0.53±0.75°C). The optimal insonation treatment time was 1 hour. APRP measurements showed a lysis threshold of 515.5±113.4 kPa. Application of 10 MHz achieved optimal lysis and lysis radius, while simultaneously proving to be the best frequency for morphologic imaging of the clot and surrounding tissue.

**Conclusions:**

These promising data provide the basis for an individualized minimal invasive ICH therapy by rtPA and sonothrombolysis independent of ICH age.

## Introduction

Spontaneous intracerebral haemorrhage (ICH) represents 10–15% of all strokes and is a major cause for death and disability in industrialized countries. As the incidence of ICH is increasing in recent years novel therapies are needed [1, 2]. A fast reduction of elevated intracranial pressure (ICP) is important to prevent further damage, once ICH occurs[3]. Reduction of blood volume and blood degradation components in fact seem to decrease secondary brain injury, especially perihematomal edema (PHE) [4]. The optimal ICH treatment strategy remains, however, a matter of debate. While the STICH-trial (surgical trial in intracerebral haemorrhage) failed to show benefits by open evacuation of deep seated hematomas in comparison to conservative therapy it did show a benefit of surgical hematoma evacuation in selected cases of lobar ICH [5–7]. A phase III trial (MISTIE III) is investigating the outcome after minimally invasive catheter-based lysis of ICH with recombinant tissue-type plasminogen activator (rtPA). Previous clinical trials have already proven the effectiveness of this method concerning volume reduction [8–10]. However, when reporting on the MISTIE II-trial, Morgan et al. noted that a sufficient hematoma volume reduction (which correlates with a better clinical outcome) could only be achieved in a small subgroup of patients despite long treatment periods [4]. Therefore, the question arises whether catheter-based hematoma evacuation could be improved to enhance efficacy.

In our previous *in vitro* study we found that an rtPA dose of 1 mg led to optimal results, independently from the actual clot size [11]. Incubation time of 15 minutes resulted in a relative end weight of approximately 50% after a single rtPA application [11]. These findings suggest that low rtPA doses might suffice. A lower affective rtPA dose would be preferable, as possible dose dependent cytotoxic effects of rtPA (increased perihematomal edema) have previously been described in clinical and experimental settings [12–14]. Furthermore it can be assumed that higher rtPA doses lead to a faster consumption of the rtPA binding partner plasminogen and thus render excessive rtPA doses ineffective [15]. During the last two decades a number of studies investigated the combination of systemic rtPA-fibrinolysis and transcranial ultrasound in the treatment of ischemic stroke. These studies demonstrated the potential of ultrasound for intravascular clot lysis, but also possible side effects, such as an increased brain edema and ICH [16–21]. Several studies have analysed the effectiveness of ultrasound assisted lysis by measuring acoustic peak rarefaction pressure (APRP) which is the main factor influencing sonothrombolysis [15, 17, 22]. One clinical trial showed an increased lysis rate in nine ICH patients using rtPA in combination with ultrasound (2 MHz probe with circular sonography mechanism on the tip of the catheter) [23]. This catheter provided no simultaneous ultrasound imaging. Yet higher ultrasound frequencies, like those used for high definition ultrasound imaging (up to 10 MHz) have not been tested for the lysis of ICH derived blood clots. The advantages of high frequency ultrasound are that it can be simultaneously applied for high-quality imaging in B-, Doppler- and Duplex-mode [24] and it has a smaller radius of ultrasound energy-distribution, which protects surrounding brain tissue [25]. A further point of interest is that the effectiveness of ultrasound clot lysis has not been investigated in relation to the age of blood clots.

This study systematically analysed the effectiveness of ultrasound and rtPA treatment for the lysis of blood clots *in vitro*. Different combinations of ultrasound and rtPA administration were applied in an *in vitro* clot system. The goals of our study were: (1) achieving higher overall lysis rates, (2) establishing an application of individualized sonothrombolysis using a specific frequency of ultrasound according to clot size, (3) improving lysis of old clots, (4) and monitoring of clot lysis by real-time ultrasound imaging.

## Material and methods

### In vitro clot system

The *in vitro* clot system used in this study has been previously described in detail [11]. Briefly, 25 ml of blood was collected from the cubital vein of healthy volunteers into 20 ml syringes (BD Discardit, Germany). After adding 10 IE of thrombin (bovine plasma thrombin, Sigma, Germany; final concentration 10IE/500 μl), the blood was mixed, poured in a balloon and placed in an incubator 1.5 h at 37°C (Heraeus Instruments, Germany). This procedure created a solid clot with a minimal liquid serum part, which was separated from the clot using a fine mesh. Before and after treatment clots and serum fraction were weighed separately. The resulting clot and serum was put in a tightly closed balloon again. Afterwards clots were randomized to the different treatment groups.

A lysis catheter (Smiths medical, Ashford, Kent, UK) was placed into all clots. In cases of ultrasound treatment an endosonography probe (AcuNav, Biosense Webster, 10 F) and a shortened urinary catheter (Unomedical, ConvaTec, UK, CH. 16) (6 holes with 3 mm diameter) were used. The endosonography catheter was placed through the shortened urinary catheter.

**Fig 1:**
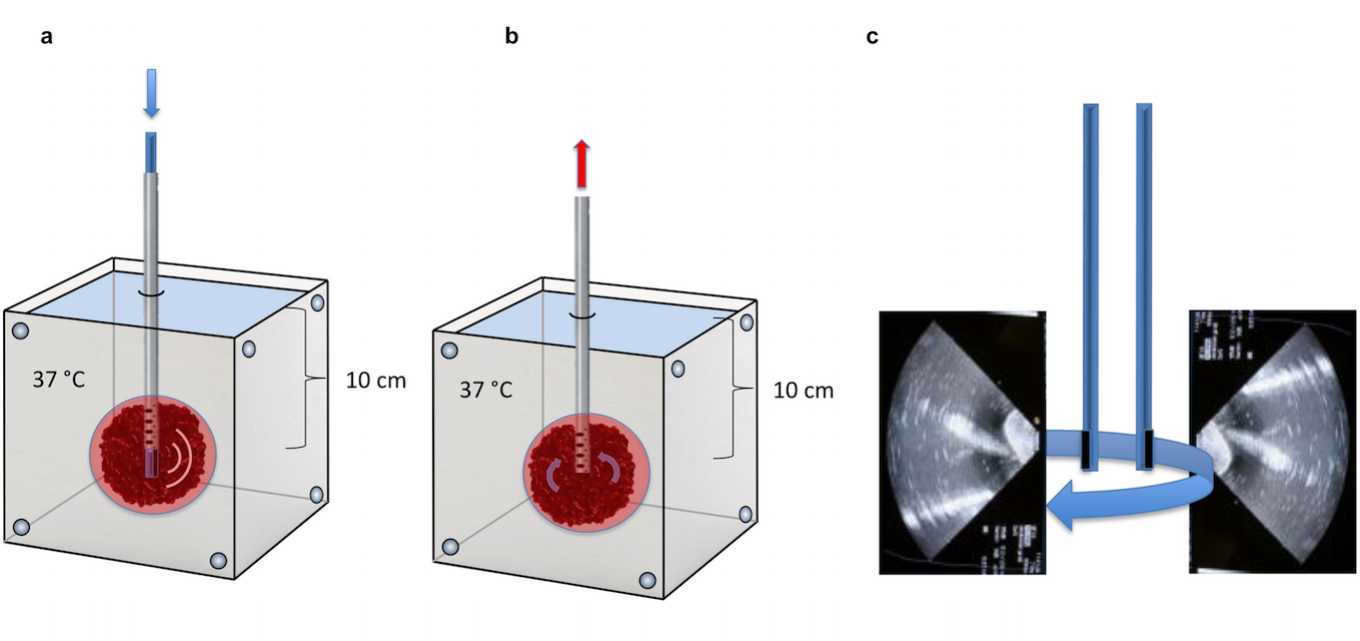
In vitro clot system. Modified figure from Keric et al. 2017. **(a)** A shortened urinary catheter (6 holes with 5 mm diameter) was placed in all clots surrounded by a balloon submerged 10 cm in a 37°C water bath. Endosonography catheter was placed through the guiding catheter and rtPA was administered. **(b)** After one hour of insonation liquefied fraction was drained by a gravity based drainage system. **(c)** Image illustrates ultrasound application: catheter with its cone shaped 22° x 90° field of view absorbing ultrasound laterally, turned in the clot after 30 min. for 180°. Ultrasound was used for one hour.

The catheter was connected to a gravity based drainage system (Neuromedex® GmbH, Switzerland). A water bath was constantly monitored by a thermometer (PH Meter, WTW GmbH, Germany) and kept at 37°C. Clot containing balloons were placed 10 cm below water surface to create the surrounding pressure equivalent to that of the intracranial cavity (Fig 1).

After completion of treatment according to the specific protocols of each group, the drainage system was opened and the liquefied blood was drained. As mentioned, after treatment clots and serum fraction were weighed separately again. We defined relative clot weight as weight after treatment in percent of clot weight before treatment.

### Sonothrombolysis

A 10 F endosonography probe (AcuNav, Biosense Webster**®**) was used in combination with the ACUSON SEQUOIA™ 512 ULTRASOUND SYSTEMS Siemens. The probe was placed via the drainage catheter in the clot core for sonothrombolysis. The endosonography probe has a cone shaped field of view (22° x 90°) absorbing ultrasound laterally. Ultrasound treatment was performed for 1h, while after 30 min it was turned 180° in order to reach a maximum field of the clot (Fig 1). Different ultrasound frequencies were investigated (10 MHz, 8.5 MHz, 7.5 MHz, 5.5 MHz). Furthermore the lowest possible mechanical index (MI) was adjusted on our device (10 MHz: MI 0.55; 8.5 MHz: MI 1; 7.5 MHz: MI 1.1; 5.5 MHz: MI 1).

### Comparison of spontaneous lysis, rtPA lysis, sonothrombolysis and combined treatment

In the first analysis we investigated the lysis rate in different treatment groups. Group 1 (control) received no treatment (n = 10, 25ml clots); Group 2 received 1 mg rtPA (n = 10, 25 ml clots); Group 3 was treated for 1 h by 10 MHz ultrasound in B-mode (n = 10, 25ml clots); Group 4 received the combination of 1 mg rtPA and 1 h 10 MHz insonation (n = 10, 25ml clots). The insonation time of 1 h was chosen based on literature reports of transcranial sonothrombolysis [19, 26]. Furthermore the frequency of 10 MHz was selected for initial experiments because of our previous experimental experience with the endosonography probe on its imaging potential [24]. Drains were opened after 1 h treatment (treatment groups and controls alike) for gravity-propelled drainage for 15 min. Then we compared all groups with respect to relative clot end weight.

Total volume of carrier (0,9%NaCl) and rtPA was 5 ml to exclude possible effects of different carrier volumes.

### Morphological analysis

Following the experiments remaining blood clots of each group (n = 3) (control, rtPA, ultrasound, ultrasound+rtPA) were fixed with 4% paraformaldehyde solution, embedded in paraffin, sliced and stained with haematoxylin and eosin (H&E) (n = 5 images per clot).

For fluorescence imaging of the fibrin mesh 5 ml blood clots were produced as described above with one difference. Before incubation for 90 min, 24h or 48h (n = 3), 0.5 mg of fibrinogen from human plasma, Alexa Fluor® 488 conjugate (Thermo Fisher Scientific, Germany) (0.5 mg/0.33ml) was added to 5 ml of blood. After the treatment the liquid part was separated from the clot by a fine mesh. For fluorescence imaging no drainage was used in order to save the structure of the clot. Immediately after treatment the clots were fixed in a 4% paraformaldehyde solution (4°C).

Confocal microscopy was performed (n = 2–10 images per clot) using a Zeiss CLSM710-NLO with Zen2009 software. All images, randomly chosen, were collected using identical settings and magnification.

### Effectiveness of ultrasound and rtPA+ ultrasound lysis for clots of different age

Clots of different age were prepared as described above adding different incubation times (1.5 h, 24h and 48h; three clots per group). These clots were insonated as described above for 1 h. This was repeated three times. The same procedure was performed with additional administration of 1 mg rtPA (n = 3). After each treatment round the liquefied part was drained using gravity-propelled drainage for 10 min. All clots (n = 18) were weighed before and after each treatment cycle.

### Optimal treatment time

To investigate the optimal treatment time for clot lysis, clots were treated with ultrasound (10 MHz) or ultrasound in combination with rtPA (1 mg) for 5, 15 and 30 minutes respectively (number of clots for each group n = 3). The clots of the control group (n = 3) were incubated in the water bath for each time periods.

Following half of the insonation time ultrasound probe was turned 180° insonating the opposite side of the clot. After treatment the lysis catheter system was opened for 15 min for gravity propelled drainage. Relative weight was assessed following treatment. The relative weights after the different treatment times were compared to the values of the 60 min treatment (n = 10) of the previous experiment.

### Lysis radius of different ultrasound frequencies

Blood clots were submerged 10cm deep in a 37°C water bath and treated for 1 h with ultrasound. The ultrasound probe was positioned in varying distances from the clot (0–8 cm in 1 cm steps, clots per group n = 3–6). 0 cm distance to probe was defined when the endosonography probe was placed in the clot core. The endosonocatheter was turned 180° after 30 min (Fig 1). Placing the ultrasound catheter directly adjacent to the clot was defined as a 1 cm distance. In this distance the probe was put on the opposite side of the clot after 50% of the insonation time because of the cone shape (22° x 90°) distribution of ultrasound probe. In further distances (2–8 cm) the clot was completely insonated.

These experiments were repeated for varying ultrasound frequencies (B-Mode: 10, 8.5, 7.5, 5.5 MHz). All clots were weighed before and after treatment and the relative end weight of the solid part was compared to each other and to the control group (clot with drainage only, n = 3). Control clots achieved a relative end weight of 70%. Therefore the lysis amount of 30% was defined as autolysis. When clots located distant to the ultrasound probe only reached a relative end weight of 70% no therapeutic effect was attributed to insonation and the distance from probe to clot was then interpreted as the lysis radius or perimeter of the lysis.

### Acoustic peak rarefaction pressure (APRP) measurement in ultrasound application

To investigate the distribution of ultrasound in space the acoustic peak rarefaction pressure (APRP) was measured in a brain phantom, consisting of 3.5 l agarose gel (Sigma Aldrich, Germany) [27]. The endosonography probe was placed in the phantom and APRP was assessed in 1 cm steps till a maximum distance of 8 cm to the probe by piezoelectric pressure transducers for high frequency measurements (PVDF Transducer M60-3L, Dr. Müller Instruments, Germany) and an oscilloscope (DSO-1062D digital-oscilloscope, Voltcraft, Switzerland). These measurements were applied to different ultrasound frequencies (B-Mode: 10, 8.5, 7.5, 5.5 MHz) and repeated three times by 2 independent investigators. Additionally the whole set of measurements was repeated in a 37°C water bath. Every value was related to a baseline measurement by a hydrophone (Müller-Platte Needle Probe M60-1, M60-3, Dr. Müller Instruments, Germany).

### Temperature recording during insonation

Temperature monitoring was performed in the brain phantom described above. Spatial temperature changes were recorded in 1 cm steps from 0 till 8 cm distance to the ultrasound probe by a thermometer (PH Meter, WTW GmbH, Germany) every 10 minutes (max 1 h). Two independent investigators assessed the values applying different ultrasound frequencies (B-Mode: 10, 8.5, 7.5, 5.5 MHz) three times (n = 3).

### Statistical analysis

Statistical analyses were performed with GraphPad Prism (version 6.0). Comparative analyses were performed by One Way Analysis of Variance and for post-hoc comparisons report adjusted p values according to the Tukey -Kramer multiple comparisons procedure. 95% confidence intervals for all parameters were reported. Two-sided P-values below 0.05 were considered as statistically significant. Results were reported as mean ± standard deviation. We performed all pairwise posthoc comparisons according to the Tukey-Kramer method and reported adjusted p-values.

For statistics of lysis radius of different ultrasound frequencies we used a non-linear regression by a three-parameter logistic model and estimated the top-value of the upper plateau with 95% confidence intervals based on the fitted model. Each top value was defined as lysis threshold. The top values are reported as mean ± standard error.

For statistical analysis of the lysis radius of different ultrasound frequencies we fitted a non linear regression by a three-parameter logistic model and estimated the top-value of the upper plateau with 95% confidence intervals based on the fitted model.

## Results

### Comparison of spontaneous lysis, rtPA lysis, sonothrombolysis and combined treatment

**Fig 2:**
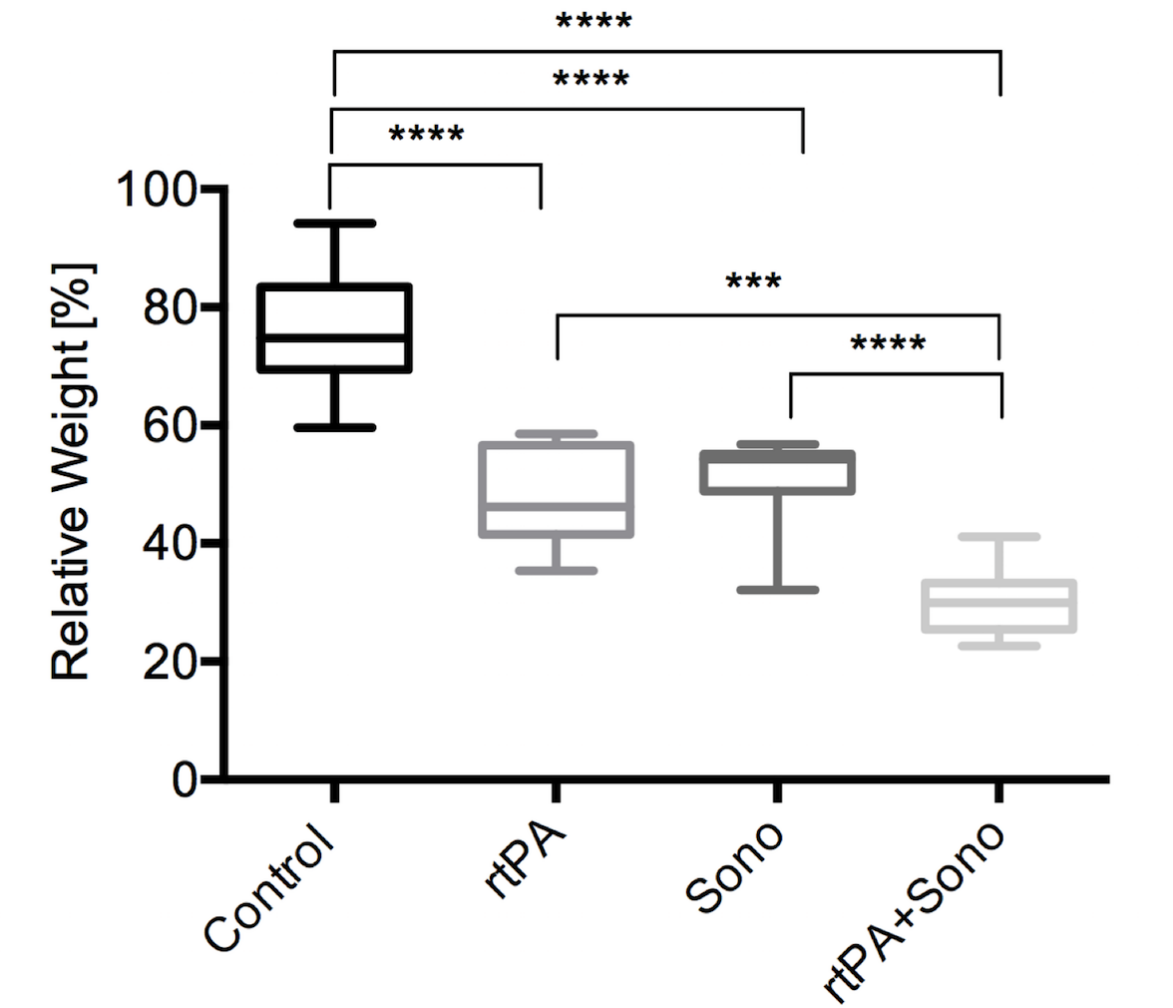
Comparison of spontaneous thrombolysis, rtPA lysis, ultrasound lysis and combined treatment. Y-axis shows the relative weight in percent of the clots (n = 10) after different treatments illustrated as box plots. The control group: blood clots treated with drain only had a relative end weight of 75.7±10.6%. Blood clots treated with 1 mg of rtPA: 47.6±7.9. Clots treated with ultrasound for one hour had a relative end weight after treatment of 51.1±7.3%. Clots treated combined with 1 mg of rtPA and one hour of ultrasound insonation (10 MHz) had the lowest relative end weight of 30.3±5.5%. **** marks the significant end weight difference of p<0.0001; *** marks the significant end weight difference of p = 0.0001. P values were adjusted according to Tukey’s multiple comparison procedure.

The control group without treatment had a relative mean end weight of 75.7±10.6%, which shows an autolysis after 1 h of incubation. Clots treated with 1 mg rtPA (group 2) showed a relative end weight of 47.6±7.9, clots treated with ultrasound for 1 hour (group 3) had a relative end weight of 51.1±7.3%, and clots treated with a combination of 1 mg rtPA+ 1 hour ultrasound (group 4) showed the significantly lowest relative end weight of 30.3±5.5% (p**≤**0.0001) compared to all other groups. All treatment groups had a significant lower relative weight compared to control group (p<0.0001). Ultrasound alone and rtPA alone achieved similar lysis rates (Fig 2, S1 Table).

### Morphological analysis

**Fig 3:**
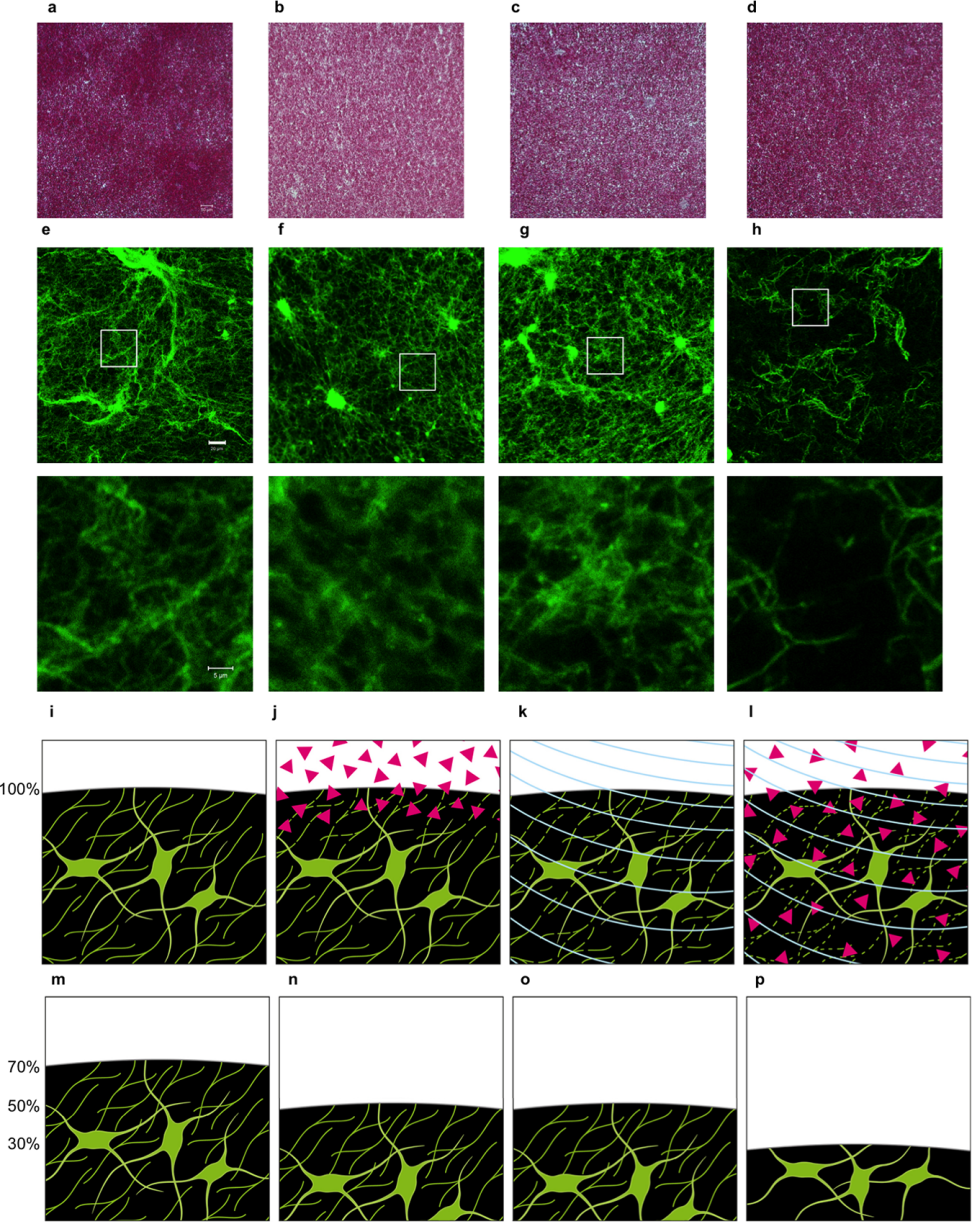
Morphological analysis. First panel: Representative HE staining of clots (n = 3) (**a)** without treatment (control) (**b)** treated with rtPA (**c)** treated with ultrasound (10 MHz) (**d)** after combined treatment (rtPA+ultrasound) showed a homogeneous structure independent of treatment mode. The bar represents 50 μm. Second panel: Representative confocal microscopic fluorescent images (n = 2–6) (fibrinogen from human plasma, Alexa Fluor® 488 conjugate) (**e)** without treatment (control) (**f)** treated with rtPA (**g)** treated with ultrasound (10 MHz) (**h)** after combined treatment (rtPA+ultrasound). The bar represents 20 μm. The white square shows the area of the zoomed image, which is shown under each image. In these zoomed images the white bar represents 5 μm. Third panel: Illustration of clots adapted to confocal imaging. Clots are illustrated (**i)** without treatment (control) (**j)** while treatment with rtPA (red triangels) (**k)** while treatment with ultrasound (blue arcs) (**l)** while combined treatment (rtPA+ultrasound). Fibrin is illustrated green. On the left side relative end weight of clots is shown in percent, starting at 100% relative end weight before treatment. (**j)** Fibrinolysis of rtPA is restricted to the surface of the clot. (**k)** Ultrasound reversible disaggregates crosslinked fibrin fibers (cutted fine, green fibers) (**l)** rtPA reaches the inside of the clot by ultrasound induced acoustic streaming. Fourth panel: Post-treatment clots. On the left side relative weight levels are shown in percent, figurative represented by clot height. (**m)** After one hour of incubation without treatment the clot has not changed stucturally, autolysis leads to a relative weight of 70%. (**n)** After treatment with rtPA (**o)** and after treatment with ultrasound relative weight is approximately 50%. Fibrin fiber structure after ultrasound treatment went back to its status before treatment. (**p)** The combined treamtent led to a relative end weight of approximately 30% and to an irreversible rarefaction of the fine fibrin mesh inside the clot.

The histomorphological analysis of H&E stained clots revealed no structural changes between the different treatment groups (Fig 3A–3D). Especially no cavitations were seen as a potential side effect of the insonation.

The laser scanning confocal microscopy images revealed no difference between the control clot, the rtPA treated clot, and the ultrasound treated clot. Only the combined treatment resulted in a rarefaction of fine fibrin fibers between robust fibrin formations (Fig 3E–3H and 3I–3P).

### Effectiveness of ultrasound and rtPA+ ultrasound in clots of different age

**Fig 4:**
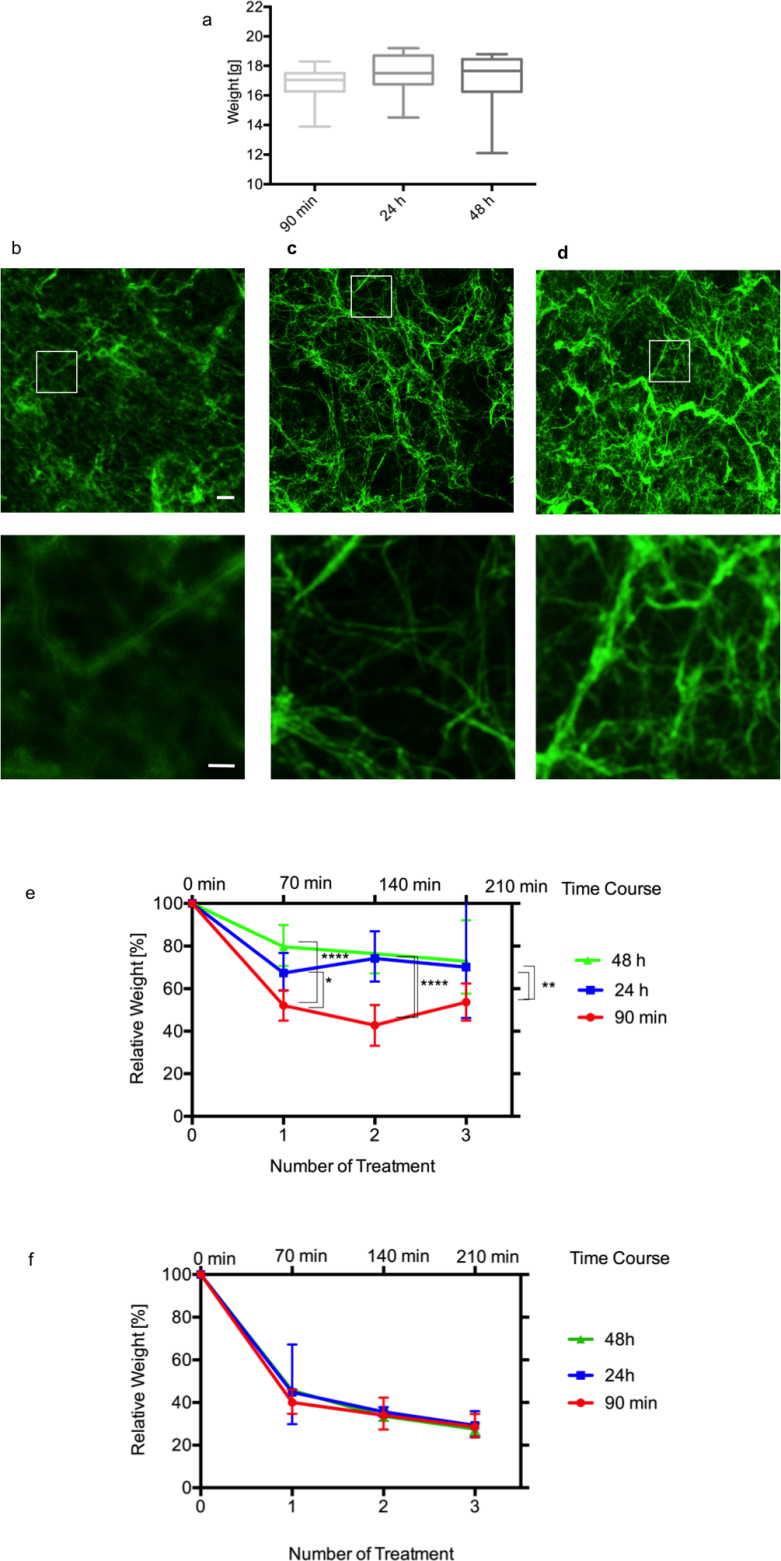
Effectiveness of ultrasound and combined treatment in clots of different age. **(a)** The Y-axis shows the absolute clot weights in gram (n = 18) after different times of incubation without any treatment. Absolute weights are illustrated in box plots. Clots of different ages: 90 min, 24 h and 48 h had an absolute weight of 16.8±1.15 g; 17.48±1.45 g; 17.14±1.7 g. **(b-d)** Representative confocal microscopic fluorescent images (n = 3–10) of 90 min **(b)**, 24 h **(c)** and 48 h **(d)** old clots (n = 3) are shown. The toolbar represents 20 μm. The white square represents the area of the zoomed image, which is shown under each image. In these zoomed images the toolbar represents 5 μm. **(e)** Clots of different ages were used: 90 min (red), 24 h (blue), 48 h (green). Y-axis shows the relative weight in percent. X-axis shows the number of treatment cycles (3 clots per condition). Time course on top shows cycles of 70 min, resulting of 1 h of ultrasound-exposure and 10 min drainage period. Significance is shown with *(p = 0.0188), **(p = 0.0085) third cycle 90 min vs. 24h clots and ** (p = 0.0028) third cycle 90 min vs. 48h clots and **** (p<0.0001). P values were adjusted according to Tukey’s multiple comparison procedure. **(f)** Effectiveness of combined treatment (ultrasound+1 mg rtPA) in clots of different age. Clot of different ages were used: 90 min (red), 24 h (blue), 48 h (green). Y-axis shows the relative end weight in percent. X-axis shows 1–3 treatment cycles (n = 3). Time course on top shows cycles of 70 min, resulting of 1 h of ultrasound and rtPA-exposure and 10 min drainage period. There are no significant differences between the different aged clots.

The clot weight did not differ significantly from 90 min, 24 h and 48 h aged clots (16.8±1.15 g; 17.48±1.45 g; 17.14±1.7 g, mean and standard deviation) (Fig 4A).

The confocal microscopic fluorescent images of the 48 h old clots tended to a more compact structure of the fibrin fibers compared to 90 min and 24 h old clots (Fig 4B–4D). Insonation alone is significantly less effective in old clots. Relative end weight of 90 min clots was significantly lower compared to 24 h (p = 0.0188) and compared to 48 h clots (p<0.0001) during the first treatment. Repetitive treatments demonstrated a decreasing lysis rate. The first treatment showed the highest impact on lysis, while the second and third treatment cycle resulted in low lysis rates in 24 h and 48 h clots compared to 90 min clots (Fig 4E, Table 1, S2 Table).

**Table 1:** Relative end weight after treatment with 1 h of ultrasound (10 MHz) in different old clots: 90 min, 24 h, 48 h.

Treatment cycles	90 min	24 h	48 h
1	52.1±7.2%	67.4±3.5%	79.8±3.9%
2	42.8±9.6%	74.3±4.7%	76.5±4%
3	53.7±8.7%	70.8±12.3%	73.1±7%

Figures are mean ± standard deviation, three clots per condition.

The combined treatment is most effective in aged clots. Interestingly there are no significant differences in old clots compared to fresh clots in every treatment cycle (Fig 4F, Table 2, S3 Table). Here also the first treatment is most effective. Lysis of old clots (24 h, 48 h) with the combined treatment was significantly better compared to lysis of old clots with ultrasound alone in every treatment cycle (p<0.0001). In contrast to ultrasound alone the combination therapy lead to higher lysis rates during repetitive treatments, but similar to other treatment modalities, the first treatment cycle seems to be the most effective. After the third combined treatment 90 min clots, 24 h and 48 h clots are significantly lighter compared to 90 min clots treated with ultrasound alone (p<0.0001).

**Table 2:** Relative end weight after combined treatment with 1 h of ultrasound (10 MHz) and 1 mg rtPA in different aged clots: 90 min, 24 h, 48 h.

Treatment cycles	90 min	24 h	48 h
1	40±2.3%	45.2±7.7%	45.6±1.2%
2	34.1±3%	35.6±0.9%	33.6±2.3%
3	28.7±2.2%	29.4±2.4%	27.5±2.7%

Figures are mean ± standard deviation, three clots per condition.

### Optimal treatment time

**Fig 5:**
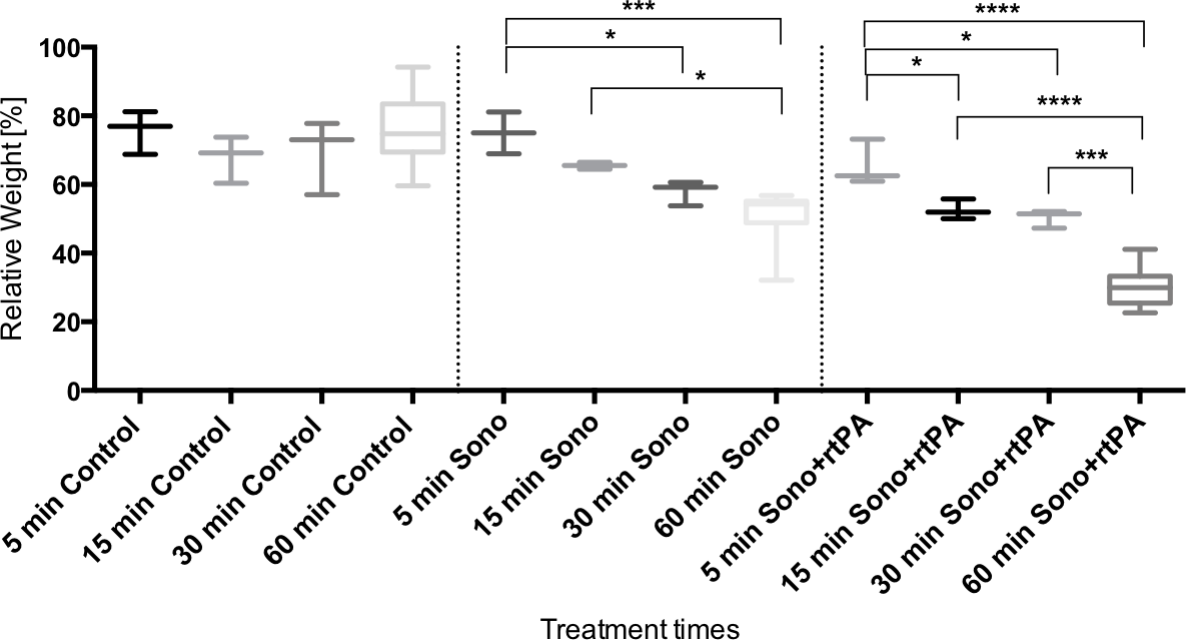
Optimal treatment time. Y-axis shows the relative clot weight (n = 3–10) in percent after different time exposures (x-axis) of the blood clots without treatment (control), to ultrasound (10 MHz) and to the combined treatment (ultrasound+rtPA) for 5, 15, 30 and 60 min. Box plots are shown, representing n = 3 clots, except the box plots of the 60 min treatment time (n = 10). Concerning the ultrasound treatment significant differences are shown (from left to right) by: * marks p = 0.0002, *** marks p = 0.019 and * marks p = 0.014. The combined treatment (ultrasound+rtPA) achieves best results of 30.3±5.5% relative end weight during one hour of treatment time. Significant differences are marked (from left to right) by: * marks p = 0.034; * marks p = 0.012; ****marks p<0.0001; *** p = 0.0002. P values were adjusted according to the Tukey-Kramer multiple comparison procedure.

The control group showed a relative weight of approximately 70% independent from treatment time. The isolated treatment with ultrasound for one hour showed significantly better results compared to the treatment for 5 min (p = 0.0002) and compared to the treatment with a 15 min time span (p = 0.014). The treatment for 30 min. showed significantly better results compared to the treatment during 5 min. (p = 0.019). Approximately 85% of lysis can be achieved during a treatment period of 30 min, assuming a maximum lysis of 100% was reached after 1 h. The combined treatment (ultrasound+rtPA) reached the best result of 30.3±5.5% relative end weight with a treatment period of one hour, significantly better than the 5 and 15 min lasting treatment (p<0.0001) and the 30 min lasting treatment (p = 0.0002). The group treated for 5 min showed the significantly worst lysis compared to the 15 min treatment period (p = 0.034) and compared to the 30 min treatment period (p = 0.012) (Fig 5, Table 3, S4 Table). The combined treatment showed a clear superiority for the one-hour treatment time. For comparative reasons we continued with a treatment time of one hour with respect to the isolated use of ultrasound although 30 min seems to be sufficient.

**Table 3:** Relative end weights after different treatment time periods.

Treatment time [min]	Control	Sono	rtPA+Sono	n
5	75.6±6.3%	75±6.1%	65.5±6.7%	3
15	67.8±6.8%	65.5±1%	52.6±3%	3
30	69.3±10.9%	57.9±3.6%	50.3±2.6%	3
60	67±11%	51.1±7.3%	30.3±5.5%	10

Figures are mean ± standard deviation, three clots per condition.

### Lysis radius of different ultrasound frequencies

**Fig 6:**
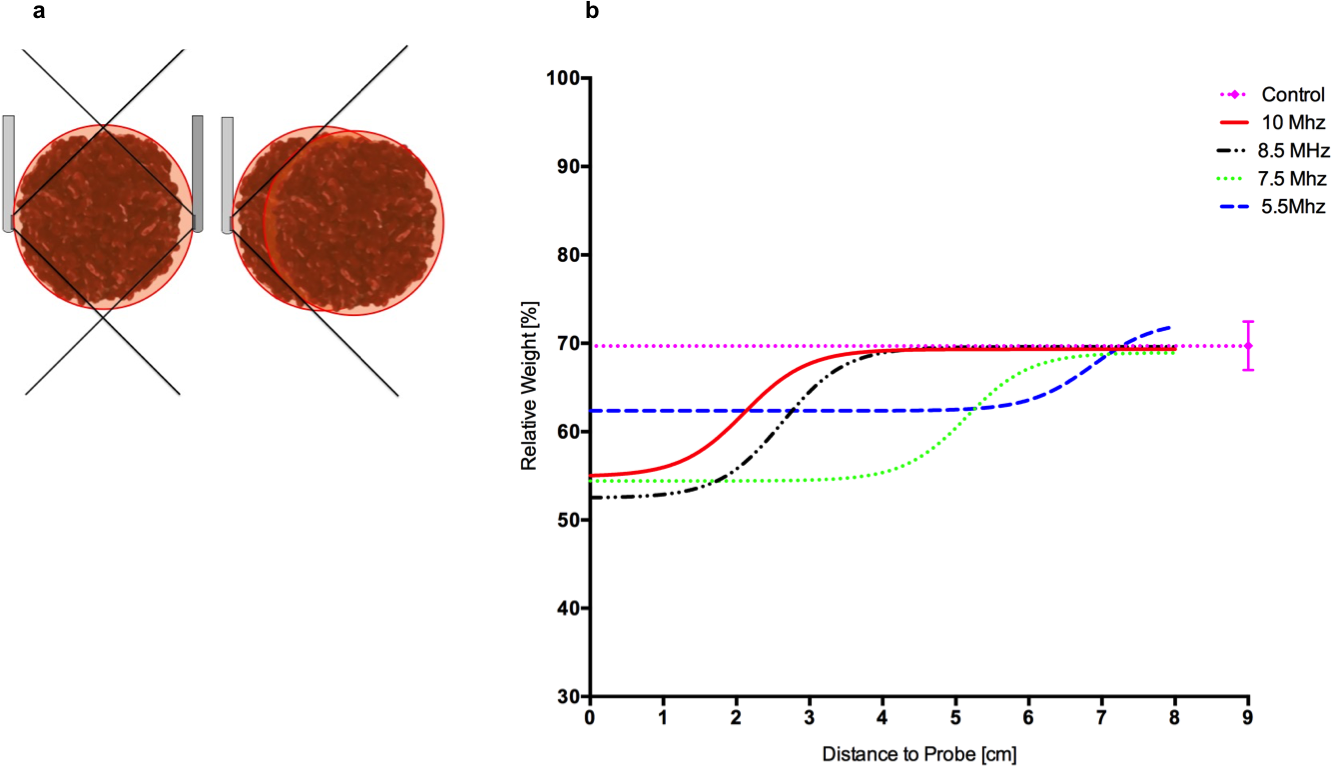
Lysis radius of different ultrasound frequencies. **(a)** Left image: For an insonation with a distance of 0 cm, the ultrasound catheter was placed inside the clot. 1 cm was defined, when the ultrasound catheter was placed directly adjacent to the clot. After 30 minutes the clot was treated from the opposite side to minimise parts of the clot not being treated because of geometric constellation of the field of view (cone shaped 22° x 90°). Right image: When the clot was treated by ultrasound from a distance of 2 cm (1cm beside the clot), the clot was reached completely by field of view and treated for one hour from one side. Further insonation distances were 0 to 8 cm with 1 cm intervals. **(b)** Relative end weight of clots (n = 3–6) (y-axis) after treatments with different ultrasound frequencies (10 MHz red, 8.5 Mhz black, 7.5 MHz green, 5.5 MHz blue, control group pink) in different distances 0 to 8 cm to probe (x-axis). A fitted non-linear regression by a three-parameter logistic model is shown. The top-value was 69.3±2.3% using 10 MHz, 69.6±4.2% using 8.5 MHz, 69±4.4% using 7.5 MHz and 72.6±10.7% using 5.5 MHz. These values correspond well with the control group wich was treated with a drainage only for one hour and had a relative weight of 69.7±2.8% (mean ± standard deviation). The mean of this relative weight was defined as a lysis threshold (pink line) without therapy effect. Consequently the lysis radius of each frequency is the corresponding x-value of the top value (10 MHz: 5 cm, 8.5 MHz: 6 cm, 7.5 MHz: 8 cm, 5.5 MHz >8 cm).

Relative end weight of the control group was 69.7±2.8% (mean standard deviation) hence approximately 30% is attributed to autolysis. Sonothrombolysis (one hour) with different frequencies (10 MHz, 8.5 MHz, 7.5 MHz, 5.5 MHz) reaches a top value of effectiveness of approximately 70% relative weight with different distance to probe (Fig 6, Table 4). These top values are in line with the relative end weight of the control group. A fitted non-linear regression by a three-parameter logistic model was used. For every frequency the top value of the curve was defined as a threshold, where the lysis radius was determined (10 MHz: 69.3±2.3%, 8.5 MHz: 69.6±4.2%, 7.5 MHz: 68.9±4.4%, 5.5: 72.6±10.7% MHz). Consequently lysis radius of 10 MHz was averaged 5 cm, of 8.5 MHz 6 cm, of 7.5 MHz 8 cm and of 5.5 MHz >8cm.

**Table 4:** Relative weight after insonation of the clots from different distances for 1 h with different frequencies.

Distance to Probe [cm]	10 MHz	8.5 MHz	7.5 MHz	5.5 Mhz	Control group without insonation
0	53.9±2.3%	45.1±6.8%	49.5±3%	60±1.6%	69.7±2.8%
1	58.4±2.7%	61.1±14.8%	62±6.1%	62.7±3.1%	
2	61.8±2.1%	55±7.7%	47.4±9.5%	56.7±7.2%	
3	66±2.3%	65.6±2.3%	55±6.6%	67.7±3.9%	
4	64±2.1%	65.5±10.2%	60.5±3.7%	61.3±6.8%	
5	69.1±5.6%	67.3±2.5%	58.9±3.8%	64.7±3.7%	
6	70.8±7.9%	70.3±4.8%	67.9±2.8%	64.7±7.7%	
7	70.3±4.3%	74.6±5.8%	68.1±7.1%	67.8±11.6%	
8	73.8±8.3%	71.4±6.9%	69.8±3.9%	72.2±6.1%	

Figures are mean ± standard deviation, 3–6 clots per condition.

### Acoustic peak rarefaction pressure (APRP) measurement in ultrasound application

**Fig 7:**
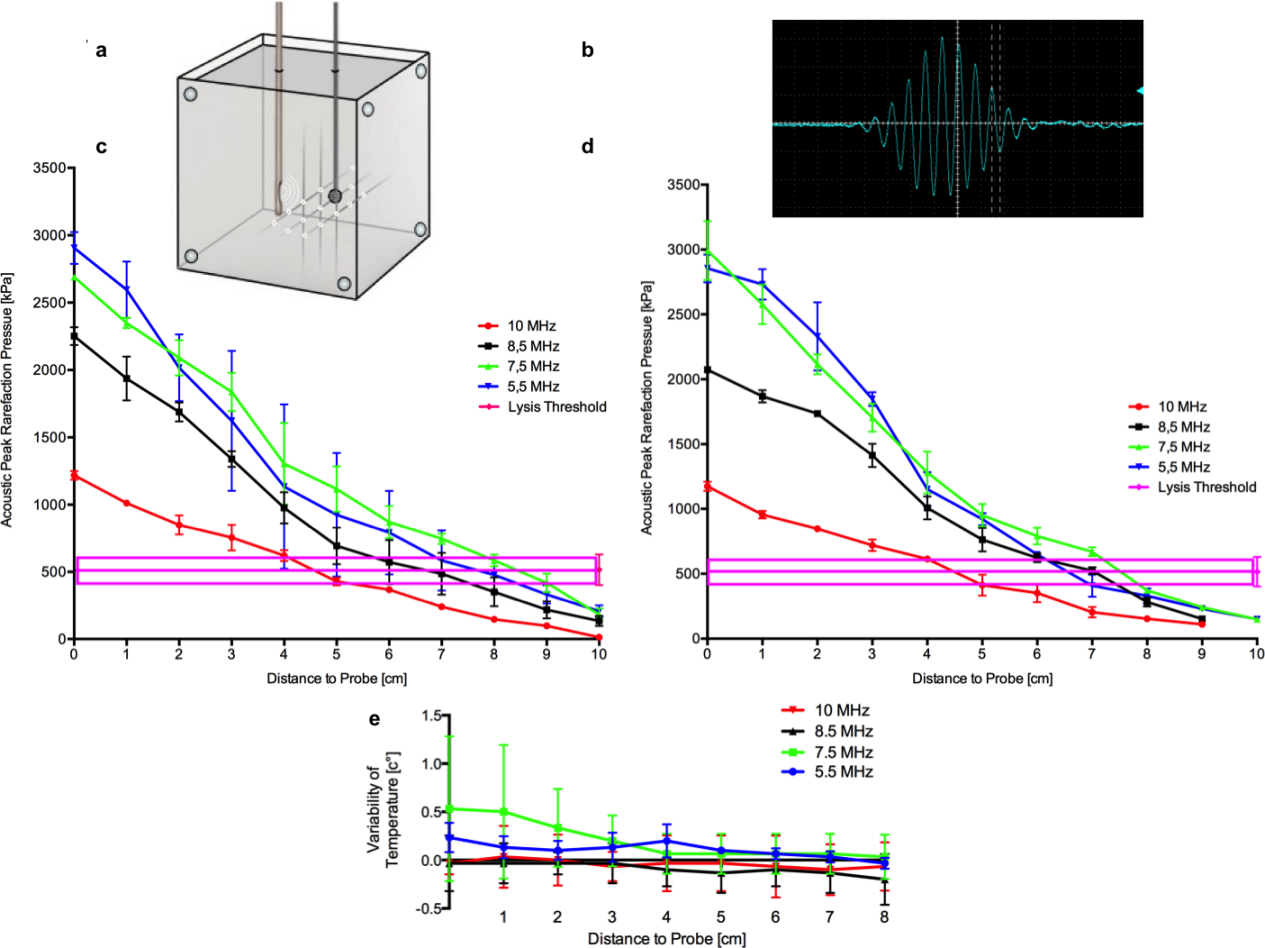
Acoustic peak rarefaction pressure (APRP) and temperature measurement in ultrasound application. **(a)** Agar brain model (3,5l). On the left side ultrasound catheter is shown, on the right side a thermometer measuring temperature from 0 till 8 cm in 1 cm steps. This was repeated with a piezoelectric pressure transducers measuring APRP from 0 till 8 cm distance to probe. APRP measurement was repeated as descibed in a water bath. **(b)** APRP signal. Largest amplitude was measured. **(c)** APRP measurement (n = 3) of ultrasound in a 37°C water bath. The mean ± standard error of APRP [kPa] (y-axis) in ralation to different distance to probe (x-axis) is shown. Different ultrasound frequencies were measured (10 MHz red, 8.5 Mhz black, 7.5 MHz green, 5.5 MHz blue). Lysis threshold was 515.5±113.4 kPa (three pink lines, mean ± standard error). Lysis threshold consists of n = 3 APRP measurements at the outer edge of lysis radius of each frequency defined in Fig 6B (10 MHz 5 cm, 8.5 MHz 6 cm, 7.5 and 5.5 MHz 8cm). **(d)** APRP measurement of ultrasound in agar. Repeated exactly as in the water bath. Values didn’t differ significantly with the values measured in the water bath. Here also 5.5 MHz showed the most unlinear measurments with the highest standard deviation. 10 and 8.5 MHz could not be measured in agar in a distance of 10 cm. **(e)** In the agarose brain model temperature variability [C°] (y-axis) was measured by thermometer every from 0–8 cm disance to probe (x-axis), every 10 min till 1 hour. Mean ± standard deviation are shown. Measurement was started at room temperature and repeated three times (n = 3). Different frequencies were measured (10 MHz red, 8.5 Mhz black, 7.5 MHz green, 5.5 MHz blue). During this time perioud ultrasound application leads to an maximal increase of temperature of 0.53±0.75 (7.5MHz).

APRP measurement showed an almost linear decrease by increasing distance to probe (Fig 7, Tables 5 and 6). Measurements in the water bath did not differ significantly from measurements in the agar brain phantom. As supposed higher frequencies showed less APRP and lower lysis radius. The frequency of 5.5 MHz showed the most nonlinear measurments with the highest standard deviation. The mean APRP defining the lysis threshold was 516±113 kPa (n = 12 APRP-measurements at the outer edge of the lysis radius of each frequency: 10 MHz, 5 cm: 428±29 kPa; 8.5 MHz, 6 cm: 572±164 kPa; 7.5 and 5.5 MHz 8cm: 585±44 kPa; 477±125 kPa). Assuming that there is one lysis threshold for each frequency 10 Mhz ultrasound has a lysis radius of 4.5±0.5cm, 8.5 Mhz has a lysis radius of 6.5±0.5cm, 7.5 Mhz of 8.5±0.5cm and 5.5 Mhz of 7.5±0.5cm (Fig 7C, Table 7). These values correspond with the lysis radius detected by insonation of blood clots.

**Table 5:** APRP measurement in a water bath with different ultrasound frequencies and with different distances to probe.

Distance to Probe [cm]	10 MHz [kPa]	8.5 MHz [kPa]	7.5 MHz [kPa]	5.5 Mhz [kPa]	Lysis threshold [kPa]
10	12.78±22.13	134.44±36.77	181.86±44.81	206.37±43.95	515.5±113.4
9	98.2±18.66	217.15±63.46	418.33±69.29	332.19±67.78	
8	145.54±10.29	350.62±107.44	**584.62±43.94**	**477.33±124.93**	
7	239.58±19.06	486.18±155.56	746.37±39.82	585.12±223.5	
6	368.52±22.62	**571.92±163.52**	870.75±120.35	792.13±310.45	
5	**428.32±29.44**	693.59±136.58	1116.17±168.86	924.42±460.63	
4	621.27±40.31	976.36±116.43	1304.27±304.37	1134.16±610.9	
3	754.27±95.64	1339.29±58.89	1836.99±142.83	1623.05±519.63	
2	849.11±71.83	1688.48±70.06	2090.06±130.73	2017.02±247.66	
1	1011.9±13.49	1936.76±162.93	2348.72±39.13	2595.41±208.92	
0	1218.27±31.92	2251.15±66.24	2689.61±16.68	2905.96±118.03	

Figures are mean ± standard error, three measurements per condition, lysis threshold of each frequency in bold.

**Table 6:** APRP measurement in an agar with different ultrasound frequencies and with different distances to probe.

Distance to Probe [cm]	10 MHz [kPa]	8.5 MHz [kPa]	7.5 MHz [kPa]	5.5 Mhz [kPa]	Lysis threshold [kPa]
10	0±0	0±0	148.84±14.56	149.87±23.11	515.5±113.4
9	110.86±5.33	152.94±3.56	242.25±24.89	230.96±8.15	
8	153.97±18.74	282.28±34.61	371.59±21.63	329.5±57.69	
7	204.27±39.59	523.5±26.85	669.27±33.92	407.51±85.13	
6	352.09±71.12	623.07±32.93	790.4±64.11	647.71±18.56	
5	412.65±80.78	762.68±90.12	950.53±86.3	919.73±46.22	
4	613.83±22.27	1006.98±88.02	1278.99±162.52	1151.71±131.66	
3	719.56±43.7	1412.44±90.17	1703.96±108.15	1847.66±53.33	
2	845.82±24.89	1734.75±17.77	2114.55±77.49	2330.11±261.91	
1	955.65±29.26	1868.2±47.04	2576.47±151.9	2730.45±116.59	
0	1173.27±35.51	2073.49±17.78	2992.2±228.55	2853.62±108.14	

Figures are mean ± standard error, three measurements per condition.

**Table 7:** Averaged lysis radius of different frequencies after measurements in a water bath.

10 MHz [cm]	8.5 MHz [cm]	7.5 MHz [cm]	5.5 Mhz [cm]
4.5±0.5	6.75±0.5	8.5±0.5	7±0.5

(mean ± standard deviation)

### Temperature measurement in ultrasound application

Application of ultrasound led to a maximum temperature increase of 0.53±0.75°C with 7.5MHz directly next to the ultrasound probe (Fig 7E, Table 8).

**Table 8:** Temperature changes during 1 hour measured in agar with different ultrasound frequencies and with different distances to probe.

Distance to Probe [cm]	10 MHz [°c]	8.5 MHz [°c]	7.5 MHz [°c]	5.5 Mhz [°c]
0	-0.03±0.12	-0.03±0.29	0.53±0.75	0.23±0.15
1	0.03±0.32	-0.03±0.21	0.5±0.69	0.13±0.12
2	-0.2±0.26	-0.03±0.12	0.33±0.4	0.1±0.1
3	-0.07±0.15	-0.03±0.21	0.2±0.26	0.13±0.15
4	-0.03±0.29	-0.1±0.17	0.07±0.21	0.2±0.17
5	-0.03±0.29	-0.13±0.21	0.07±0.21	0.1±0
6	-0.07±0.32	-0.1±0.17	0.07±0.21	0.07±0.06
7	-0.1±0.26	-0.13±0.21	0.07±0.21	0.03±0.06
8	-0.07±0.25	-0.2±0.26	0.03±0.23	-0.03±0.06

Figures are mean ± standard error, three measurements per condition.

## Discussion

Although several trials have investigated rtPA lysis in ICH [8, 9, 13, 28] and one clinical trial used combined rtPA and sonothrombolysis in ICH-patients [23] systematic studies to determine optimal rtPA doses and especially ultrasound modalities are lacking. One of the reasons for this might be the absence of standardized, highly reproducible clot models. While introducing a simple, highly reliable *in vitro* clot model in our recent paper [11] we applied this model here for establishing clear relations between clot lysis by rtPA in combination with the use of different ultrasound modalities (such as frequency and distance of the probe). Besides we could investigate the effect of clot age (in vitro) on the effectiveness of clot lysis therapies. The answers found using the *in vitro* model might reduce the number of animal experiments required and help optimize conditions for further clinical trials on ICH [11, 29] as well with other experimental and clinical studies.

The application of ultrasound alone and administration of rtPA alone resulted in a similar volume reduction of app. 50%. In contrast to these groups the combination therapy (rtPA+ultrasound) achieved a significantly higher lysis rate and reached a volume reduction of app. 70% with only a single administration (Fig 2). The result of the rtPA therapy alone is in line with our previous study [11] and with other experimental and clinical studies [8, 9, 12, 23, 28, 30, 31].

The positive results obtained here to increase the fibrinolytic effect using a combination of rtPA and ultrasound leads to further questions: What would be the optimal ultrasound modalities in terms of treatment time and duration, frequency and lysis radius? Recent studies investigating recanalization of clotted arteries by ultrasound in stroke patients chose an insonation time of 1 hour [16, 18–21]. Looking at the extent of clot lysis after distinct insonation times (5, 15, 30 and 60 min) with or without the additional use of rtPA, we assumed that the maximum extend of lysis is indeed reached after about 1 hour, agreeing with the insonation time applied in the above cited studies to be effective. We found an optimal treatment time of one hour using the combined treatment (Fig 5). Although we found that approximately 85% of lysis is reached after 30 min by the isolated use of ultrasound and although we previously reported of an optimal rtPA incubation time of 15 min [11], for practical reasons and for reasons of comparability in this study rtPA was left in place during the complete insonation time, which was one hour for the combined treatment and for the isolated ultrasound use.

One *in vitro* study exists, which showed an enhancement of lysis by 10 MHz ultrasound only with additional use of ultrasound contrast agents (UCA) and rtPA [32]. An explanation could be the short insonation time of 10 min.

The only clinical study on combined treatment (rtPA+ intralesional ultrasound) on ICH so far used 2 MHz ultrasound without the possibility of intracranial imaging [23]. This could have however practical reasons, as 2 MHz is a common frequency regarding the transcranial application of ultrasound. For our experiments, focusing on the application of an intracranial ultrasound catheter, we were able to consider other ultrasound frequencies for sonothrombolysis and tested the effect of frequencies up to 10 MHz (as used for intracranial imaging [24]). 10 MHz indeed proved to be the optimal frequency in our *in vitro* experiments.

When considering optimization of clot lysis the issue of clot age comes in view. Yet no data is available on the sonothrombolytic effect in relation to the clot age. While it is clear that in vivo the tissue surrounding the clot plays a role in clot formation and lysis, the center of larger blood clots, like those frequently encountered in ICH, are less prone to these changes and in vitro data might well help to understand and optimize lysis treatment. Untreated clots of different ages (90 min, 24 h and 48 h) were analyzed to assess the autolytic potential, especially induced by endogenous plasmin. Interestingly the clot weight did not differ significantly between fresh and older clots (Fig 4A) leading to the assumption that the main process of autolysis (70–80% relative end weight) takes place in the first hours. In this in vitro-system a longer incubation time did not resulted in a higher lysis rate. A fibrinolytic treatment was necessary to reduce clot weight (Fig 4E and 4F). Similar to our previous results of the rtPA treatment [11] the ultrasound application alone was less effective in older clots (24 h and 48 h) than in fresh clots (1.5 h) (Fig 4E). The morphological analysis of 90 min, 24 h and 48 h old clots showed a more compact mesh of fibrin fibers in the 48 h old clots. We assume that lower lysis rates in old clots can be attributed to the higher density of these clots. The compact clot provides a limited surface, which can be approached by rtPA molecules. Interestingly the combination of rtPA+ultrasound resulted in same lysis rate independent from clot age (Fig 4F).

Furthermore 1 mg rtPA was equally effective independently of the clot size [11]. This phenomenon could also be the result of a limited clot surface, which could be reached by the catheter and the rtPA. The increasing enzymatic fibrinolysis with additional ultrasound use could be explained by mechanisms such as acoustic streaming [33]. There is evidence that ultrasound leads to a reversible only short-lasting disruption of cross-linked fibrin fibers. Temporary disconnection of fibrin fibers leads to a loosening of the clot density [15, 34]. Fibrinolytic agents are able to stream into the clot, connect to additional binding sites and reach an irreversible rarefaction of the fine fibrin mesh (Fig 3I–3P). The uptake of rtPA is accelerated by increased clot surface area [15, 34]. This process explains the superiority of the combined treatment, which could provide a promising minimally invasive therapy option for patients with ICH older than 24 h. As they often suffer from neurological deterioration, mass effect caused by edema, hematoma expansion or neurotoxicity of blood products in the further course of ICH [35]. Furthermore Pieters et al. described in their in vitro studies an ultrasound-induced transport of plasminogen from the outer plasma into the clot and also in transverse direction [15]. In our study the fluorescence microscopy visualized the fibrin mesh of the clot supporting the thesis of acoustic streaming. No structural differences were seen between the ultrasound treated and the untreated control clots, while combined treated clots showed a loosened fibrin mesh (Fig 3E–3H).

To further characterize the space around the endosonography probe, in which lysis is possible, clots were insonated with different frequencies (10 MHz, 8.5 MHz, 7.5 MHz, 5.5 MHz). The analysis revealed that the higher the frequency, the lower the lysis radius.

A threshold and maximum perimeter for lytic effects can also be evaluated by measuring the APRP. In the literature these APRP values are very heterogeneously reported. Values range from 30 kPa up to 2.1 MPa, while they are calculated, estimated or measured using different frequencies (40 kHz-10 MHz) and different insonation durations [15, 17, 22]. We measured APRP for each frequency separately at the outer perimeter of lysis radius and found that our values were approximately 500 kPa (mean 515.5±113.4 kPa) (Fig 7C and 7D). The question whether there is one APRP lysis threshold for other frequencies, not analysed here needs to be answered in the future by investigating other frequencies, which clearly differ from the frequencies applied during this study. The application of 10 MHz enables lysis within a radius of 4.5±0.5 cm (Figs 6B, 7C and 7D). Most common ICH of approximately 30–45 ml could be treated sufficiently using this frequency; bearing in mind that 10 MHz ultrasound provides the best imaging quality [24]. This finding is supported by the fact that for transcranial Doppler of infants with open fontanelle ultrasound frequencies of 5 to 10 MHz are preferred [35]. For larger ICH >45–60 ml [36], depending on individual geometric configuration of ICH and catheter placement, the use of 8.5 MHz ultrasound with a lysis radius of approximately 7 cm is possible (Figs 6B, 7C and 7D). Interestingly, penetration depth of 10 MHz and 8.5 MHz correspond well with the assessed lysis radius [25]. For an intracranial application a larger lysis radius of approximately 8 cm (by 7.5 and 5.5 MHz) is not necessary. The measurements of the 5.5 MHz frequency showed the highest standard deviation concerning APRP values and lysis radius was not as high as expected. Increasing inaccuracy by growing distance to the probe could be an explanation for varying APRP values, so that these results should be interpreted with caution.

Potential tissue damage by temperature enhancement has been suggested as a side effect of ultrasound application [16]. Hence we assessed temperature increase around the probe at different frequencies, distances and time periods. In our setting we found a maximum temperature increase of 0.53±0.75°C during insonation of one hour only using 7.5 MHz frequency close to the ultrasound probe. This supports the benefit of 10 and 8.5 MHz, while for the assessment of damage induced in the clot adjacent brain tissue our results have obviously to be combined with animal investigations, as any biological effects cannot be investigated in this *in vitro* model.

Adverse events of intracranial ultrasound should however not be underestimated. In a clinical trial 26 patients with middle cerebral artery occlusion underwent either rtPA therapy alone or rtPA+ultrasound (low-frequency transcranial ultrasound 300 kHz). The study had to be stopped prematurely, as 13 of 14 patients had signs of hemorrhage in MRI, 5 of those were symptomatic [18]. Brain edema, irritation of the blood-brain-barrier and tissue-necrosis are further possible adverse events of ultrasound applications, which should be avoided [37, 38].

For this reasons we adjusted our ultrasound probe to the lowest mechanical index (MI) (10 MHz: MI 0.55; 8.5 MHz: MI 1), as the MI is a function of APRP and frequency and thus an indicator for cavitation related bio effects [39]. Our APRP measurements did not exceed MI values on the display of our ultrasound device. A MI of 0.7 is known as the threshold for cavitation formation [40]. Apfel et al. suggested therefore in the TCD application an MI lower than 0.7 [39], which corresponded to the MI of the 10 MHz ultrasound application in our study. Cavitations which arise in liquids as vaporous bubbles, have also been discussed as a possible intensifying mechanisms for sonothrombolysis [41, 42]. Nevertheless cavitations also bear the risk for perihematomal tissue injury as mentioned above [32]. In our in vitro 10 MHz treated clots the H&E staining showed no cavities (Fig 3A–3D).

Advantages of catheter-based intralesional ultrasound application are the lower penetration depth of higher frequencies protecting surrounding tissue [25]. Compared to thrombolysis using transcranial Doppler (TCD) the use of intralesional ultrasound avoids absorption and attenuation of ultrasound waves by the bone barrier [16]. Moreover TCD application has the risk of standing waves and heating near the bone caused by the difference of impedance between bone and brain tissue [43]. An intralesional setting prevents this. In contrast to other catheter-based ultrasound systems [23] the endosonography catheter investigated in this study is suitable for simultaneous real-time imaging, which can be used for monitoring of clot size, or merged with anatomical CT or MRI scans for image-guidance [24]. In patients the application of real-time monitoring could reduce the necessity of dangerous transports of critical ill patients from intensive care unit to the CT.

### Limitations

Results from this study have to be interpreted with caution. The *in vitro* clot model does not consider the perifocal intracerebral metabolic surrounding and the intracranial pressure situation in an ICH case. All experiments were performed by maintenance a pressure of 10 cm H_2_O. Though effects of ICP pulsations connected with blood pressure variations could not be adequately reflected in this clot system. The clots were generated from blood of healthy volunteers, which excluded the influence of antiplatelet and anticoagulant treatment as major risk factors of ICH's. Furthermore the oval and round shape of the clots does not resemble all configurations of ICH's. Despite these limiting issues, the clot system provides a valuable tool for systematic investigation.

## Conclusion

Using our standardized *in vitro* clot system we could establish clear relations between clot lysis, rtPA and ultrasound modalities (alone and in combination with each other). Thus we could demonstrate the high effectiveness of catheter-based intralesional fibrinolysis with the combination of ultrasound and rtPA. Results were significantly better than in the control, ultrasound or rtPA group. One hour insonation using a high ultrasound frequency of 10 MHz in combination with rtPA was found to be safe, most effective and has an optimal lysis radius for intracranial use. Our findings need to be confirmed in a large animal model of ICH to evaluate biological effects on the clot and the surrounding brain tissue. The optimized treatment modalities determined in this study might however reduce the number of animals required and help to optimize future clinical trials.

## Compliance with ethical standards

Ethical approval: This study was approved by the local Ethical Committee of Rhineland Palatinate. All blood samples were taken after informed consent of healthy volunteers.

## Supporting information

S1 Table Clot weights after spontaneous lysis, rtPA lysis, sonothrombolysis and combined treatment. (DOCX)

S2 Table Clot weights after treatment with 1 h of ultrasound (10 MHz) in different old clots: 90 min, 24 h, 48h. (DOCX)

S3 Table Clot weights after combined treatment with 1 h of ultrasound (10 MHz) and 1 mg rtPA in different aged clots: 90 min, 24 h, 48 h. (DOCX)

S4 Table Clot weights after different treatment time periods. (DOCX)

## References

[1] Kreitzer N, Adeoye O. An update on surgical and medical management strategies for intracerebral hemorrhage. Semin Neurol. 2013 11;33(5):462–7. doi: 10.1055/s-0033-1364210 24504609 10.1055/s-0033-1364210

[2] Mozaffarian D, Benjamin EJ, Go AS, Arnett DK, Blaha MJ, Cushman M, et al Heart disease and stroke statistics—2015 update: a report from the American Heart Association. Circulation. 2015 1 27;131(4):e29–322. doi: 10.1161/CIR.0000000000000152 25520374 10.1161/CIR.0000000000000152

[3] Fernandes HM, Siddique S, Banister K, Chambers I, Wooldridge T, Gregson B, et al Continuous monitoring of ICP and CPP following ICH and its relationship to clinical, radiological and surgical parameters. Acta Neurochir Suppl. 2000;76:463–6. 11450068 10.1007/978-3-7091-6346-7_96

[4] Fiorella D, Gutman F, Woo H, Arthur A, Aranguren R, Davis R. Minimally invasive evacuation of parenchymal and ventricular hemorrhage using the Apollo system with simultaneous neuronavigation, neuroendoscopy and active monitoring with cone beam CT. J Neurointerventional Surg. 2015 10;7(10):752–7. 10.1136/neurintsurg-2014-011358 25186443

[5] Mendelow AD, Gregson BA, Fernandes HM, Murray GD, Teasdale GM, Hope DT, et al Early surgery versus initial conservative treatment in patients with spontaneous supratentorial intracerebral haematomas in the International Surgical Trial in Intracerebral Haemorrhage (STICH): a randomised trial. The Lancet [Internet]. 2005 1 [cited 2015 Nov 6];365(9457):387–97. Available from: http://linkinghub.elsevier.com/retrieve/pii/S014067360517826X 10.1016/S0140-6736(05)17826-X 15680453

[6] Mendelow AD, Gregson BA, Rowan EN, Murray GD, Gholkar A, Mitchell PM, et al Early surgery versus initial conservative treatment in patients with spontaneous supratentorial lobar intracerebral haematomas (STICH II): a randomised trial. Lancet Lond Engl. 2013 8 3;382(9890):397–408. 10.1016/S0140-6736(13)60986-1 PMC3906609 23726393

[7] Gregson BA, Broderick JP, Auer LM, Batjer H, Chen X-C, Juvela S, et al Individual patient data subgroup meta-analysis of surgery for spontaneous supratentorial intracerebral hemorrhage. Stroke J Cereb Circ. 2012 6;43(6):1496–504. 10.1161/STROKEAHA.111.640284 PMC3419479 22511006

[8] Lippitz BE, Mayfrank L, Spetzger U, Warnke JP, Bertalanffy H, Gilsbach JM. Lysis of basal ganglia haematoma with recombinant tissue plasminogen activator (rtPA) after stereotactic aspiration: initial results. Acta Neurochir (Wien). 1994;127(3–4):157–60. 7942196 10.1007/BF01808759

[9] Morgan T, Zuccarello M, Narayan R, Keyl P, Lane K, Hanley D. Preliminary findings of the minimally-invasive surgery plus rtPA for intracerebral hemorrhage evacuation (MISTIE) clinical trial. Acta Neurochir Suppl. 2008;105:147–51. 19066101 10.1007/978-3-211-09469-3_30

[10] Abdu E, Hanley DF, Newell DW. Minimally invasive treatment for intracerebral hemorrhage. Neurosurg Focus. 2012 4;32(4):E3 doi: 10.3171/2012.1.FOCUS11362 22463113 10.3171/2012.1.FOCUS11362

[11] Keric N, Masomi-Bornwasser J, Müller-Werkmeister H, Kantelhardt SR, König J, Kemski O, et al Optimization of Catheter Based rtPA Thrombolysis in a Novel In Vitro Clot Model for Intracerebral Hemorrhage. BioMed Res Int [Internet]. 2017 3 26 [cited 2017 Mar 29];2017:e5472936 Available from: https://www.hindawi.com/journals/bmri/2017/5472936/abs/10.1155/2017/5472936 PMC5385248 28459065

[12] Rohde V, Rohde I, Thiex R, Ince A, Jung A, Dückers G, et al Fibrinolysis therapy achieved with tissue plasminogen activator and aspiration of the liquefied clot after experimental intracerebral hemorrhage: rapid reduction in hematoma volume but intensification of delayed edema formation. J Neurosurg. 2002 10;97(4):954–62. doi: 10.3171/jns.2002.97.4.0954 12405387 10.3171/jns.2002.97.4.0954

[13] Thiex R, Tsirka SE. Brain edema after intracerebral hemorrhage: mechanisms, treatment options, management strategies, and operative indications. Neurosurg Focus. 2007;22(5):E6 17613237 10.3171/foc.2007.22.5.7

[14] Keric N, Maier GS, Samadani U, Kallenberg K, Dechent P, Brueck W, et al Tissue plasminogen activator induced delayed edema in experimental porcine intracranial hemorrhage: reduction with plasminogen activator inhibitor-1 administration. Transl Stroke Res. 2012 7;3(Suppl 1):88–93. doi: 10.1007/s12975-012-0188-3 23538320 10.1007/s12975-012-0188-3 PMC3605490

[15] Pieters M, Hekkenberg RT, Barrett-Bergshoeff M, Rijken DC. The effect of 40 kHz ultrasound on tissue plasminogen activator-induced clot lysis in three in vitro models. Ultrasound Med Biol. 2004 11;30(11):1545–52. doi: 10.1016/j.ultrasmedbio.2004.08.028 15588966 10.1016/j.ultrasmedbio.2004.08.028

[16] Pfaffenberger S, Devcic-Kuhar B, Kollmann C, Kastl SP, Kaun C, Speidl WS, et al Can a commercial diagnostic ultrasound device accelerate thrombolysis? An in vitro skull model. Stroke J Cereb Circ. 2005 1;36(1):124–8. 10.1161/01.STR.0000150503.10480.a7 15591211

[17] Barlinn K, Tsivgoulis G, Molina CA, Alexandrov DA, Schafer ME, Alleman J, et al Exploratory analysis of estimated acoustic peak rarefaction pressure, recanalization, and outcome in the transcranial ultrasound in clinical sonothrombolysis trial. J Clin Ultrasound JCU. 2013 8;41(6):354–60. doi: 10.1002/jcu.21978 22927038 10.1002/jcu.21978

[18] Daffertshofer M, Gass A, Ringleb P, Sitzer M, Sliwka U, Els T, et al Transcranial low-frequency ultrasound-mediated thrombolysis in brain ischemia: increased risk of hemorrhage with combined ultrasound and tissue plasminogen activator: results of a phase II clinical trial. Stroke J Cereb Circ. 2005 7;36(7):1441–6. 10.1161/01.STR.0000170707.86793.1a 15947262

[19] Alexandrov AV, Demchuk AM, Felberg RA, Christou I, Barber PA, Burgin WS, et al High Rate of Complete Recanalization and Dramatic Clinical Recovery During tPA Infusion When Continuously Monitored With 2-MHz Transcranial Doppler Monitoring. Stroke [Internet]. 2000 3 1 [cited 2017 Jan 8];31(3):610–4. Available from: http://stroke.ahajournals.org/content/31/3/610 10700493 10.1161/01.str.31.3.610

[20] Cintas P, Traon APL, Larrue V. High Rate of Recanalization of Middle Cerebral Artery Occlusion During 2-MHz Transcranial Color-Coded Doppler Continuous Monitoring Without Thrombolytic Drug. Stroke [Internet]. 2002 2 1 [cited 2017 Jan 8];33(2):626–8. Available from: http://stroke.ahajournals.org/content/33/2/626 11823681 10.1161/hs0202.103073

[21] Eggers J, Koch B, Meyer K, König I, Seidel G. Effect of ultrasound on thrombolysis of middle cerebral artery occlusion. Ann Neurol. 2003 6;53(6):797–800. doi: 10.1002/ana.10590 12783427 10.1002/ana.10590

[22] Soltani A, Singhal R, Obtera M, Roy RA, Clark WM, Hansmann DR. Potentiating intra-arterial sonothrombolysis for acute ischemic stroke by the addition of the ultrasound contrast agents (Optison^TM^ & SonoVue(®)). J Thromb Thrombolysis. 2011 1;31(1):71–84. doi: 10.1007/s11239-010-0483-3 20473551 10.1007/s11239-010-0483-3 PMC2990803

[23] Newell DW, Shah MM, Wilcox R, Hansmann DR, Melnychuk E, Muschelli J, et al Minimally invasive evacuation of spontaneous intracerebral hemorrhage using sonothrombolysis. J Neurosurg. 2011 9;115(3):592–601. doi: 10.3171/2011.5.JNS10505 21663412 10.3171/2011.5.JNS10505 PMC3785332

[24] Keric N, Kantelhardt SR, Neulen A, Dechent P, Henning A, Vollmer FC, et al Image-guided intracranial endosonography. J Neurosurg Anesthesiol. 2013 7;25(3):317–23. doi: 10.1097/ANA.0b013e31828cb27e 23552276 10.1097/ANA.0b013e31828cb27e

[25] Schäberle W. Ultrasonography in Vascular Diagnosis: A Therapy-Oriented Textbook and Atlas. Springer Science & Business Media; 2005. 364 p.

[26] Bor-Seng-Shu E, Nogueira RDC, Figueiredo EG, Evaristo EF, Conforto AB, Teixeira MJ. Sonothrombolysis for acute ischemic stroke: a systematic review of randomized controlled trials. Neurosurg Focus. 2012 1;32(1):E5 doi: 10.3171/2011.10.FOCUS11251 22208898 10.3171/2011.10.FOCUS11251

[27] Linninger A, Basati S, Dawe R, Penn R. An Impedance Sensor to Monitor and Control Cerebral Ventricular Volume. Med Eng Phys [Internet]. 2009 9 [cited 2015 Oct 26];31(7):838–45. Available from: http://www.ncbi.nlm.nih.gov/pmc/articles/PMC2752330/ doi: 10.1016/j.medengphy.2009.03.011 19419900 10.1016/j.medengphy.2009.03.011 PMC2752330

[28] Schaller C, Rohde V, Meyer B, Hassler W. Stereotactic puncture and lysis of spontaneous intracerebral hemorrhage using recombinant tissue-plasminogen activator. Neurosurgery. 1995 2;36(2):328–33; discussion 333–5. 7731513 10.1227/00006123-199502000-00012

[29] Wagner KR, Xi G, Hua Y, Kleinholz M, de Courten-Myers GM, Myers RE, et al Lobar intracerebral hemorrhage model in pigs: rapid edema development in perihematomal white matter. Stroke. 1996 3;27(3):490–7. 8610319 10.1161/01.str.27.3.490

[30] Thiex R, Rohde V, Rohde I, Mayfrank L, Zeki Z, Thron A, et al Frame-based and frameless stereotactic hematoma puncture and subsequent fibrinolytic therapy for the treatment of spontaneous intracerebral hemorrhage. J Neurol. 2004 12;251(12):1443–50. doi: 10.1007/s00415-004-0554-5 15645342 10.1007/s00415-004-0554-5

[31] Wagner KR, Xi G, Hua Y, Zuccarello M, de Courten-Myers GM, Broderick JP, et al Ultra-early clot aspiration after lysis with tissue plasminogen activator in a porcine model of intracerebral hemorrhage: edema reduction and blood-brain barrier protection. J Neurosurg. 1999 3;90(3):491–8. doi: 10.3171/jns.1999.90.3.0491 10067918 10.3171/jns.1999.90.3.0491

[32] Mizushige K, Kondo I, Ohmori K, Hirao K, Matsuo H. Enhancement of ultrasound-accelerated thrombolysis by echo contrast agents: dependence on microbubble structure. Ultrasound Med Biol. 1999 11;25(9):1431–7. 10626631 10.1016/s0301-5629(99)00095-2

[33] Braaten JV, Goss RA, Francis CW. Ultrasound reversibly disaggregates fibrin fibers. Thromb Haemost. 1997 9;78(3):1063–8. 9308755

[34] Francis CW, Blinc A, Lee S, Cox C. Ultrasound accelerates transport of recombinant tissue plasminogen activator into clots. Ultrasound Med Biol. 1995;21(3):419–24. 7645133 10.1016/0301-5629(94)00119-x

[35] 1115—transcranial.pdf [Internet]. [cited 2017 Jan 10]. Available from: http://www.aium.org/resources/guidelines/transcranial.pdf

[36] Lord AS, Gilmore E, Choi HA, Mayer SA. Time Course and Predictors of Neurological Deterioration after Intracerebral Hemorrhage. Stroke J Cereb Circ [Internet]. 2015 3 [cited 2017 Jan 8];46(3):647–52. Available from: http://www.ncbi.nlm.nih.gov/pmc/articles/PMC4739782/10.1161/STROKEAHA.114.007704 PMC4739782 25657190

[37] Wilhelm-Schwenkmezger T, Pittermann P, Zajonz K, Kempski O, Dieterich M, Nedelmann M. Therapeutic Application of 20-kHz Transcranial Ultrasound in an Embolic Middle Cerebral Artery Occlusion Model in Rats. Stroke [Internet]. 2007 3 1 [cited 2017 Jan 8];38(3):1031–5. Available from: http://stroke.ahajournals.org/content/38/3/1031 doi: 10.1161/01.STR.0000257966.32242.0b 17272763 10.1161/01.STR.0000257966.32242.0b

[38] Nedelmann M, Reuter P, Walberer M, Sommer C, Alessandri B, Schiel D, et al Detrimental effects of 60 kHz sonothrombolysis in rats with middle cerebral artery occlusion. Ultrasound Med Biol. 2008 12;34(12):2019–27. doi: 10.1016/j.ultrasmedbio.2008.06.003 18723268 10.1016/j.ultrasmedbio.2008.06.003

[39] Apfel RE, Holland CK. Gauging the likelihood of cavitation from short-pulse, low-duty cycle diagnostic ultrasound. Ultrasound Med Biol. 1991;17(2):179–85. 2053214 10.1016/0301-5629(91)90125-g

[40] Miller DL, Gies RA. The influence of ultrasound frequency and gas-body composition on the contrast agent-mediated enhancement of vascular bioeffects in mouse intestine. Ultrasound Med Biol. 2000 2;26(2):307–13. 10722920 10.1016/s0301-5629(99)00138-6

[41] Datta S, Coussios C-C, McAdory LE, Tan J, Porter T, De Courten-Myers G, et al CORRELATION OF CAVITATION WITH ULTRASOUND ENHANCEMENT OF THROMBOLYSIS. Ultrasound Med Biol [Internet]. 2006 8 [cited 2017 Jan 8];32(8):1257–67. Available from: http://www.ncbi.nlm.nih.gov/pmc/articles/PMC1937506/ doi: 10.1016/j.ultrasmedbio.2006.04.008 16875959 10.1016/j.ultrasmedbio.2006.04.008 PMC1937506

[42] Prokop AF, Soltani A, Roy RA. Cavitational mechanisms in ultrasound-accelerated fibrinolysis. Ultrasound Med Biol. 2007 6;33(6):924–33. doi: 10.1016/j.ultrasmedbio.2006.11.022 17434661 10.1016/j.ultrasmedbio.2006.11.022

[43] Nedelmann M, Gerriets T, Kaps M. Therapeutische Ultraschallbehandlung des akuten Hirnarterienverschlusses. Nervenarzt [Internet]. 2008 12 1 [cited 2017 Jan 5];79(12):1399 Available from: http://link.springer.com/article/10.1007/s00115-008-2550-y. doi: 10.1007/s00115-008-2550-y 18704356 10.1007/s00115-008-2550-y

