# Correction: Combination of ultrasound and rtPA enhances fibrinolysis in an *In Vitro* clot system

**DOI:** 10.1371/journal.pone.0200456

**Published:** 2018-07-05

**Authors:** Julia Masomi-Bornwasser, Philipp Winter, Hendrik Müller-Werkmeister, Susanne Strand, Jochem König, Oliver Kempski, Florian Ringel, Sven R. Kantelhardt, Alf Giese, Naureen Keric

Dr. Alf Giese is not included in the author byline. He should be listed as the ninth author and affiliated with Department of Neurosurgery, University Medical Center of the Johannes Gutenberg University, Mainz, Germany and OrthoCentrum Hamburg, Hamburg, Germany. The contributions of this author are as follows: Contributed to the categories conceptualization and methodology.

The following information is missing from the Funding section: AG is a paid employee of OrthoCentrum. The specific roles of this author are articulated in the ‘author contributions’ section.
