# Supplementary material for: Combination of ultrasound and rtPA enhances fibrinolysis in an In Vitro clot system
Source: PLoS One. 2017 Nov 16;12(11):e0188131. doi: 10.1371/journal.pone.0188131 (PMC5690612; doi:10.1371/journal.pone.0188131)
Supplement: S1 Table — (DOCX) [file pone.0188131.s001.docx]

**Table S1. Clot weights after spontaneous lysis, rtPA lysis, sonothrombolysis and combined treatment.**

| Control | rtPA | Sono | rtPA+Sono | n |
| --- | --- | --- | --- | --- |
| 16.63±2.03 g | 10.17±1.7 g | 11.3±1.93 g | 6.74±1.31 g | 10 |

(mean ± standard deviation).
