# Supplementary material for: Combination of ultrasound and rtPA enhances fibrinolysis in an In Vitro clot system
Source: PLoS One. 2017 Nov 16;12(11):e0188131. doi: 10.1371/journal.pone.0188131 (PMC5690612; doi:10.1371/journal.pone.0188131)
Supplement: S2 Table — (DOCX) [file pone.0188131.s002.docx]

**Table S2. Clot weights after treatment with 1 h of ultrasound (10 MHz) in different old clots: 90 min, 24 h, 48 h.**

| Treatment cycles | 90 min | 24 h | 48 h |
| --- | --- | --- | --- |
| 1 | 8.67±2.23 g | 11.5±0.89 g | 13.07±1.63 g |
| 2 | 7.06±1.17 g | 11.99 ±0.6 g | 9.68±3.28 g |
| 3 | 7.66±2.23 g | 9.6±3.82 .g | 9.79±3.76 g |

(n=3), (mean ± standard deviation).
