# Supplementary material for: Combination of ultrasound and rtPA enhances fibrinolysis in an In Vitro clot system
Source: PLoS One. 2017 Nov 16;12(11):e0188131. doi: 10.1371/journal.pone.0188131 (PMC5690612; doi:10.1371/journal.pone.0188131)
Supplement: S3 Table — (DOCX) [file pone.0188131.s003.docx]

**Table S3. Clot weights after combined treatment with 1 h of ultrasound (10 MHz) and 1 mg rtPA in different aged clots: 90 min, 24 h, 48 h.**

| Treatment cycles | 90 min | 24 h | 48 h |
| --- | --- | --- | --- |
| 1 | 6.77±0.24 g | 8.05±1.61g | 8.1±0.5 g |
| 2 | 5.78±0.59 g | 6.31±0.46 g | 5.97±0.93 g |
| 3 | 4.96±0.51 g | 5.58±0.52 g | 4.94±0.3 g |

(n=3), (mean ± standard deviation).
