# Supplementary material for: Combination of ultrasound and rtPA enhances fibrinolysis in an In Vitro clot system
Source: PLoS One. 2017 Nov 16;12(11):e0188131. doi: 10.1371/journal.pone.0188131 (PMC5690612; doi:10.1371/journal.pone.0188131)
Supplement: S4 Table — (DOCX) [file pone.0188131.s004.docx]

**Table S4. Clot weights after different treatment time periods.**

| Treatment time [min] | Control | Sono | rtPA+Sono | n |
| --- | --- | --- | --- | --- |
| 5 | 12.73±1.15 g | 11.33±1.99 g | 10.63±1.65 g | 3 |
| 15 | 10.53±0.15 g | 9.7±0.69 g | 8.57±1.1g | 3 |
| 30 | 10.33±0.25 g | 9.1±0.44 g | 8.1±1.04g | 3 |
| 60 | 14.52±4.13g | 11.3±1.93 g | 6.89±1.34 g | 10 |

(mean ± standard deviation).
